# Tuberculosis presenting as multiple intramuscular nodules in a child: a case report

**DOI:** 10.1186/s13256-015-0543-6

**Published:** 2015-03-28

**Authors:** Ajaya Kumar Dhakal, Subhash Chandra Shah, Devendra Shrestha, Niroj Banepali, Geetika KC

**Affiliations:** 1 Department of Paediatrics, KIST Medical College Teaching Hospital, Imadol, Lalitpur, Nepal; 2 Department of Surgery, KIST Medical College Teaching Hospital, Imadol, Lalitpur, Nepal; 3 Department of Pathology, KIST Medical College Teaching Hospital, Imadol, Lalitpur, Nepal

**Keywords:** Intramuscular nodules, Skeletal muscle tuberculosis, Tubercular muscular nodules, Tuberculosis

## Abstract

**Introduction:**

Tuberculosis is a global health problem that mostly affects people in developing countries. Tuberculosis can occur in various organ systems and may present with diverse manifestations in the same system. Primary muscular tuberculosis is a very rare condition in both adults and children, and tuberculosis of various muscle groups presenting as intramuscular nodules is an even more uncommon presentation.

**Case presentation:**

A 9-year-old Asian girl presented with multiple painless, gradually progressive swellings over different parts of her body for 3 months with no history of contact with tuberculosis. A physical examination was normal except for multiple swellings in her right forearm, a single swelling in her interscapular region and multiple swellings in her right calf. Ultrasonography of swellings revealed multiple nodules in the intramuscular layer. Excisional biopsies performed from two different sites revealed swellings in muscular layers and histopathology showed granulomatous inflammation with caseous necrosis consistent with tuberculosis. The child was started on antitubercular therapy after which the swellings resolved; she was kept on regular follow up.

**Conclusions:**

Intramuscular nodules in multiple muscular sites may be the presenting symptoms of tuberculosis of the muscles. Tuberculosis of skeletal muscles should be considered in a differential diagnosis when presented with single or multiple masses even when a chest X-ray is normal and there is no evidence of tubercular foci elsewhere in the body.

## Introduction

Tuberculosis is a public health problem throughout the world and a significant cause of mortality and morbidity in both adults and children in developing as well as developed nations [1]. The large number of tuberculosis-related deaths and morbidity can be prevented with early diagnosis and timely intervention with antitubercular therapy [1]. Tuberculosis can affect most organs/systems of the human body and its clinical manifestations in each system are diverse. Sometimes tuberculosis occurs at unusual sites with unusual presentations simulating other medical illness causing diagnostic difficulties [2]. A high index of suspicion and early detection of these uncommon types of tuberculosis may lead to better outcomes.

Tuberculosis of muscles is a very rare form of extrapulmonary tuberculosis which manifests with different presentations such as isolated muscle mass [3], tubercular myositis [4], isolated muscular abscess [5] and tubercular pyomyositis [6]. Tuberculosis of muscles presenting as intramuscular nodules in different parts of the body is an extremely rare form of muscular tuberculosis and hence reported here.

## Case presentation

**Figure 1:**
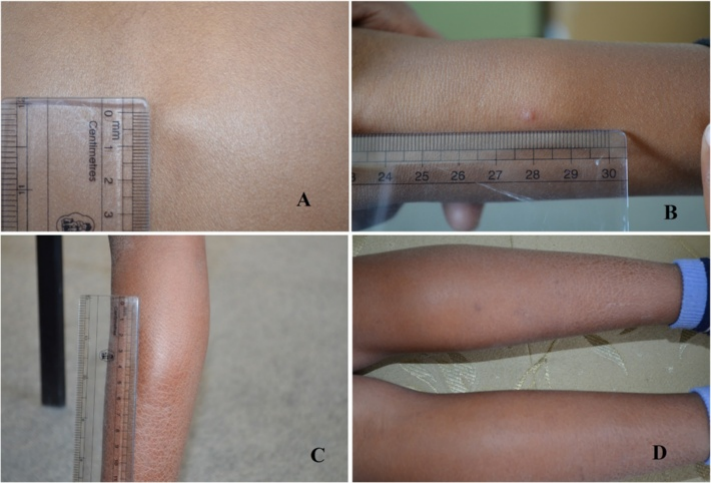
**Photograph of swellings. A**. Interscapular region. **B**. Forearm. **C** and **D**. Right calf.

A 9-year-old Asian girl presented to our paediatric clinic with complaints of multiple swellings in her forearm, calf and back for 3 months which were painless and gradually progressive in nature. There was no history of fever, trauma, joint pain, rashes, prolonged cough, decreased appetite, weight loss as well as no known or traceable history of contact with tuberculosis. On examination her weight and height were appropriate for her age. There were no signs of pallor, icterus, generalized lymphadenopathy, rashes, discharging sinus or features suggestive of osteomyelitis and joint involvement. The systemic examination was unremarkable except for presenting swellings. There was a small swelling in her back which was 1.5cm×1.5cm (Figure 1A), swellings in her right forearm (Figure 1B) and multiple swellings in her right calf (Figure 1C and D) of varying size which were soft, raised, nontender, mobile, not fixed with skin and had normal overlying skin.

**Figure 2:**
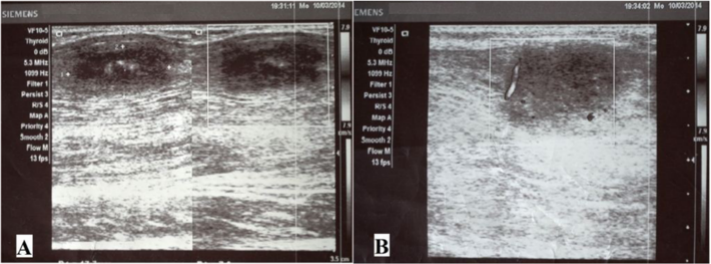
**Ultrasonography of swellings. A**. Well-defined hypoechoic nodules measuring 1.7cm×2.4cm were seen in the calf muscle. There were associated echogenic foci seen in the centre of the lesion suggesting calcification. **B**. On colour Doppler minimal peripheral vascularity was noted.

**Figure 3:**
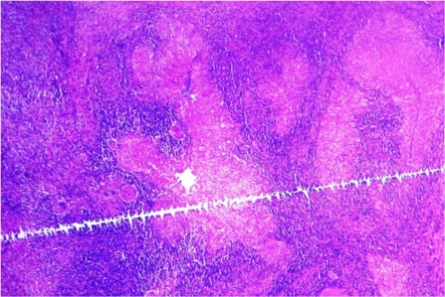
**Photomicrograph of excisional biopsy of nodule from interscapular region.** Several well-formed granulomas embedded among the muscle fibres (hematoxylin and eosin stain, ×50).

**Figure 4:**
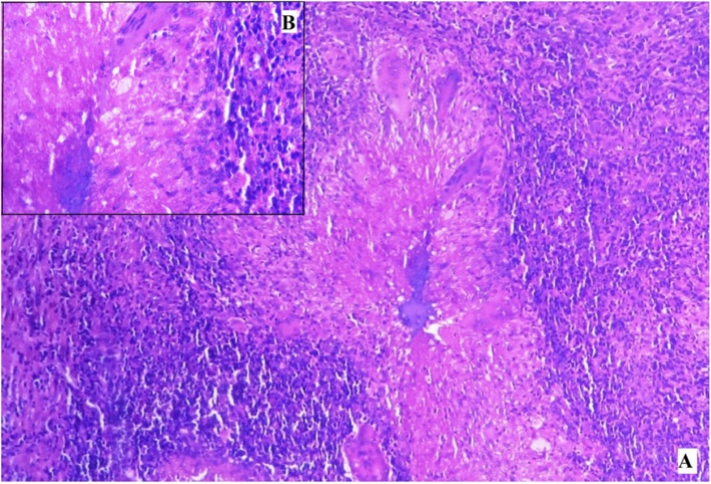
**Photomicrograph of excisional biopsy of nodule from interscapular region. A**. Giant cells and epithelioid granuloma (hematoxylin and eosin stain, ×100). **B**. Inset showing central caseous necrosis surrounded by epithelioid cells, lymphocytes and plasma cells in higher magnification (hematoxylin and eosin stain, ×400).

**Figure 5:**
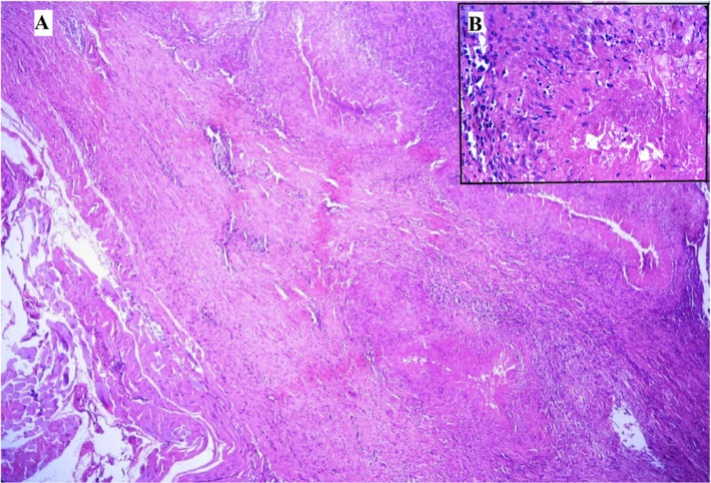
**Photomicrograph of excisional biopsy of nodule from forearm. A**. Several well-formed granulomas with caseous necrosis (hematoxylin and eosin stain, ×50). **B**. Inset showing central caseous necrosis surrounded by epithelioid cells, lymphocytes and plasma cells (hematoxylin and eosin stain, ×400).

**Figure 6:**
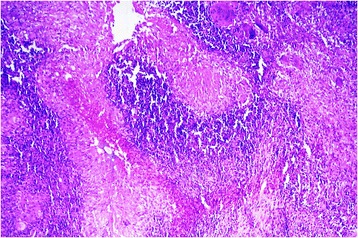
**Photomicrograph of excisional biopsy of nodule from forearm.** Giant cells and epithelioid granuloma (hematoxylin and eosin stain, ×100).

On investigation her haemoglobin was 11.1gm/dL with normocytic normochromic red blood cells morphology. Her platelet count was 320000/mm^3^ and total white blood cells count was 8300/mm^3^ with neutrophil 66%, lymphocytes 29%, and eosinophils 5%. Her erythrocyte sedimentation rate was 26mm/hour. There were no atypical cells and parasites in a peripheral smear. The results of renal, liver function tests and stool examination were normal. A radiological investigation showed normal chest X-ray and limbs radiography for osteomyelitis was negative and there were no calcifications in chest and limb radiographs. A Mantoux test revealed no induration after 72 hours. Ultrasonography of her swellings revealed well-defined hypoechoic intramuscular lesions of which the largest measured 2.4cm×1.7cm and there were also a few calcifications and cystic degeneration in some of the lesions (Figure 2). Abdominal ultrasonography revealed normal findings except for a hyperechoic space-occupying lesion on the right lobe of her liver measuring 1.6cm×1.3cm suggesting hemangioma. Ultrasound-guided fine-needle aspiration cytology (FNAC) of the swelling revealed features suggestive of parasitic cysts based on the finding of bluish fibrillary structures with interspersed small nuclei in a background of mixed inflammatory cells consisting of neutrophils, eosinophils, lymphocytes and histiocytes. Acid-fast bacilli staining of aspirate was negative for tubercular bacilli and aspirate culture was also negative. An excision biopsy of the swelling from her interscapular region was performed. Intraoperatively the swelling was located in the muscular layer and histopathology showed features of granulomatous inflammation with caseous necrosis, consistent with tuberculosis (Figures 3 and 4) but the result for serum cysticercus immunoglobulin G antibodies was 2.23 (positive>1.1). An excision biopsy was repeated again at a different site from her right forearm after 10 days due to dubious diagnosis which also showed swelling located in the muscular layer and it also showed features of tuberculosis in histopathology (Figures 5 and 6). However, the geneXpert MTB-RIF assay from the biopsy specimen and tissue culture of biopsy specimen for tuberculosis revealed no tubercular bacilli. There were no characteristic histopathological findings of neurocysticercosis, filarial infections, hydatid cysts of muscles and sarcoidosis on both biopsy specimens. Based on her history, clinical examination and biopsy report from two different sites showing granulomatous inflammation with caseous necrosis suggestive of tuberculosis plus high prevalence of tubercular infection, diagnosis of tubercular infection was made. She was started on an antitubercular regimen according to National Tuberculosis Programme of Nepal and all the swellings resolved during the intensive phase and she is now on the continuation phase of the antitubercular regimen.

## Discussion

Primary muscular tuberculosis is a very uncommon condition in both children and adults [7]. Current knowledge about these conditions is mainly based on case reports because the precise incidence of this disease is not known. A study found four cases of tuberculosis of muscles out of 2224 autopsies performed on patients with a history of tuberculosis [8]. Moreover another study reported one case of primary muscular tuberculosis out of 6180 patients with different forms of tuberculosis [9]. Similarly, 20 cases of muscular tuberculosis were found in a study from China over the period of 10 years of which only one case out of 20 presented with mass in multiple muscles whereas most of the patients had mass in a single muscle [10]. Tuberculosis of muscle presenting as swelling has been mostly reported to present as a mass in a single muscle [3,10]. However, recently skeletal muscle tuberculosis simultaneously involving multiple sites presenting as multiple muscle masses in different parts of the body has also been reported from India [7]. These case reports highlight the uncommon nature of these conditions.

The rare occurrence of skeletal muscle tuberculosis has been hypothesised to be due to “high lactic acid content of muscles, high vascularity and blood flow, absence of reticuloendothelial and lymphatic tissue and the highly differentiated state of muscle tissue” which inhibit the growth of *Mycobacterium* [11]. The child was diagnosed as a case of tuberculosis of muscles based on clinical examination, presence of multiple swellings in muscles on ultrasonography, intraoperative finding of swelling in muscular layer, caseating granuloma on histopathology consistent with tuberculosis and rapid clinical response to antitubercular therapy. However, there was no history of contact with tuberculosis and a Mantoux test was negative. It must be highlighted that in previous studies [3,7,10] most of the patients were diagnosed as muscular tuberculosis with muscle biopsy and very few of these cases had a positive Mantoux test. In one study of muscular tuberculosis out of 11 patients who underwent a purified protein derivative (PPD) test only one was strongly positive while the other 10 were negative [10].

Tubercular involvement of the muscles has been postulated to be the result of haematogenous dissemination of tubercular lesions on lungs [4,12], contagious transmission from an underlying structure like tuberculosis of the bones, tubercular arthritis and synovial lining [4], or without evidence of active foci of tuberculosis elsewhere in the body or osseous involvement [3] and direct traumatic inoculation [4]. The presence of multiple swellings in different parts of the body with normal examination of bone and no evidence of bone involvement in radiography makes the possibility of contagious transmission from an underlying structure less likely in this patient. There were no symptoms of cough, fever, loss of appetite and weight loss along with normal chest X-ray suggesting absence of active foci of pulmonary tuberculosis. It has been suggested that in an area with high incidence of tuberculosis, muscular tuberculosis should be considered strongly when clinical features like muscular pain and swelling are present even though a chest X-ray is normal and there is no evidence of active foci of tuberculosis elsewhere in the body [5]. The pathogenesis for multiple intramuscular swellings in this patient with no evidence of active foci of tuberculosis elsewhere could only be explained by reactivation of latent tubercular bacilli at the state of reduced immunity during the active growth period of the child, which had lodged in muscle during period of lymphohematogenous spread of primary tuberculosis [2,7].

Neurocysticercosis, hydatid cyst of muscles and filarial infections are common causes of superficial palpable multiple nodular swellings in different parts of a body [13]. These diseases were ruled out based on clinical features and histopathological finding of caseating granuloma, absence of scolex of cysticercus, absence of cyst and fragments of acellular lamellate membrane of *Echinococcus* and absence of microfilaria including absence of eosinophils in the biopsy specimens of this patient. However serum IgG antibody for cysticercus was positive, which might be due to high exposure of cysticercus parasite in the general population. One of the studies from the South East Asian region found *Taenia* cysticercus antigens in 4.5% and positive cysticercus IgG antibodies in 15.9% of the asymptomatic population [14]. The FNAC of swelling of this patient demonstrated findings suggestive of parasitic cyst which were inconclusive in determining specific parasitic infections. In a study done on 361 patients presenting with superficial nodular swelling, FNAC found 35 cases suggestive of parasitic infestation; however, only 14 of these cases could be definitely diagnosed as parasitic cyst by FNAC alone and the rest of the cases required biopsy [13]. In this patient also, final diagnosis was achieved with biopsy of swellings.

The limitation of this case report is that magnetic resonance imaging (MRI) of the soft tissue could not be performed to confirm that all the masses are from muscular tissue because of the unavailability of MRI in our centre and cost factors. However, ultrasonography and intraoperative findings were conclusive of intramuscular lesions.

## Conclusions

Tuberculosis is a common infection in the developing world and a high index of suspicion of skeletal muscle tuberculosis should be kept in mind with a patient presenting with multiple swellings in different parts of his or her body in an endemic area. The diagnosis could be achieved with history and clinical examination supported by appropriate investigations and histopathology.

## Consent

Written informed consent was obtained from the parent of the patient for publication of this Case Report and any accompanying images. A copy of the written consent is available for review by the Editor-in-Chief of this journal.
